# Bibliometric analysis of output and impact based on CRIS data: a case study on the registered output of a Dutch university

**DOI:** 10.1007/s11192-015-1788-y

**Published:** 2015-11-12

**Authors:** Thed N. van Leeuwen, Erik van Wijk, Paul F. Wouters

**Affiliations:** CWTS, Leiden University, Wassenaarseweg 62a, PO Box 905, 2300 AX Leiden, The Netherlands

**Keywords:** Research impact assessment, Bibliometric analysis, CRIS system, Non WoS citation impact analysis

## Abstract

In this study we combine the registered output of a whole university in the Netherlands with data retrieved from the Web of Science. The initial research question was: is it possible to show the impact of the university in its’ full broadness, taking into account the variety of disciplines covered in the research profile of the university? In order to answer this question, we analyzed the output of the university as registered in the CRIS system METIS, over the years 2004–2009. The registration covers a wide variety of scholarly outputs, and these are all taken into account in the analysis. In the study we conduct analyses on the coverage of the output of the university, both from the perspective of the output itself, towards the Web of Science (“external”), as well as from the Web of Science perspective itself (“internal”). This provides us with the necessary information to be able to draw clear conclusions on the validity of the usage of standard bibliometric methodologies in the research assessment of universities with such a research profile.

## Introduction

It the Netherlands research is evaluated periodically, according to the guidelines and concepts written down in the Standard Evaluation Protocol (SEP), a protocol designed and supported by the Association of Dutch universities (VSNU), the national Research Council (NWO) and the Royal Dutch Academy of Sciences (KNAW). This SEP is also the informal guideline for assessment in research environments that are not under the supervision of these three stakeholders in the Dutch research landscape.

In many assessments, peer review is complemented with a bibliometric study. The application of bibliometrics is not explicitly required by the SEP, but often the availability of quantitative data in a research assessment setting is seen as desirable, as these indicators are considered of value in the process, not only during the assessment period, but often also after the assessment had taken place. Therefore, the major natural sciences disciplines assessments (chemistry in 2010, physics in 2011, and biology in 2011) have been accompanied by bibliometric data. In some engineering disciplines, as well as psychology, bibliometric results have been supplied to the persons involved. Next, assessments of NWO and KNAW research institutes have been supplied with reports on the research performance of the organization and the levels of research within (departments, groups, etc.).

Bibliometric analyses tend to provide information on the state of the scientific impact of universities and the domains therein, as that is the main strength of bibliometrics (Moed et al. 1985). As the most used bibliometric techniques are based upon the journal literature as processed for either the Web of Science (WoS) or Scopus, it is obvious that for research fields in which journal literature, and more in particular the internationally oriented English language journal literature as processed for these two electronic data systems, play a less dominant role, the current bibliometric toolbox is insufficient and inadequate to measure scientific performance or impact (Schöpflin 1992; Hicks 1999; Luwel et al. 1999; Moed et al. 2002).

These fields, and we are talking about the humanities, the ‘softer’ parts of the social sciences (such as anthropology, political science, sociology, etc.), and law, do not use the international journal literature as their main medium to communicate research findings with their international peers (van Leeuwen 2013). So in order to be able to tell about their scientific performance or the scientific impact, we have to start with an analysis of the various types of output coming from these fields.

The study was part of an institution broad analysis on “The impact of the university”. Part of that initiative focused on other elements in impact analysis, such as the societal impact of the university, which can then be split up into economic impacts, cultural impacts, social impacts, etc. For the analysis of the impact of the scholarly activities, the aim was on the production and impact of the scholarly outputs. Given the above, we collected the data for this study from an in-house research information system, called METIS. This type of information systems tend to offer many opportunities to solve data availability in bibliometric studies (Aksnes and Revheim 2000). In this study we will use the internally registered publication data and combine these data with the Web of Science data, in order to provide as wide as possible an overview of output and impact across the disciplines covered in the university’s output. This approach is complementary to the methodology of data collection and processing described on other types of bibliometric analyses (van Leeuwen 2007).

While output registration has been organized locally in the Netherlands, internationally we find some variation in the ways the output of the scholarly system is registered. Among the countries with a national registration system, Norway has the longest tradition in national registration of research and scholarly outputs. In Norway a nationwide system for output registration called FRIDA was installed in the previous decade, which was later replaced by the current system, called CRISTiN. In this system, all entities in the system that produce scientific outputs are registering their products, from the universities to the polytechnic and applied science universities to research institutions and hospitals (Schneider 2009; Sivertsen 2010; Sivertsen and Larsen 2012). Next to this Norwegian system, Belgium, or more accurately Flanders, has experience with a registration system. In Flanders a performance based funding model has been implemented, in which a formula is used that is based on research outputs (Debackere and Glanzel 2004). As this formula is linked to the usage of Journal Impact Factor values, the social sciences and humanities communities protested as that was putting them in a disadvantageous position compared to their natural, life and biomedical sciences colleagues. Therefore, in the second part of the previous decade, in Flanders an initiative was taken to create a comprehensive data system that covers the scholarly production of the social sciences and humanities, as a support tool in research policy, and more in particular in research funding. This database system, called VABB-SHW, covers integrally the scholarly publication output of the research community in the SSH domains in Flanders (Engels et al. 2012; Verleysen and Engels 2012, Ossenblok et al. 2012, 2014). More recently, Denmark moved towards a system of performance based funding, for which purpose a national output registration was necessary. In Denmark the universities have a variety of systems to register their output in, so there the choice was made to create a platform that unifies the variation of outputs in such a way that one format can be used by the Danish Ministry for research funding allocation purposes. In all three countries a system was adopted that added a certain weight to publications not published in the international, mostly English language journal literature as covered by Web of Science or Scopus. So non covered journals, books chapters, and books are given a certain weight in the research funding allocation procedures applied.

In the Netherlands we have a long tradition of registering research outputs. The most used system has been METIS. METIS is a system that was developed at the Radboud University in Nijmegen, the Netherlands, and as such a commercial offspring from that university. The application of the system was followed quickly in the Dutch academic landscape. Some Dutch universities have been using METIS for already 15 years. The most important element of METIS is metadata, as it contains bibliographic information on a wide variety of scholarly outputs, such as journal publications, books, book chapters, but also conference proceedings, reports, theses, magazine contributions, and many more. The system is used for both output registration as well as for management purposes, as it can contain also information on journal classifications and journal metrical scores, as well as funding sources for research. Next to that, also information from the HRM department can be stored in METIS, making it a multi-purpose tool for university management purposes. Next to the institutional level, METIS can also be used for the individual researchers, as it allows for CV building. The system is filled on shop floor level, often by support staff. As such, publications can be entered into the system twice, that is, originating from different organizational units. That is not a problem in our study, as we wanted to show the differences in general between the various organization units of the university under study. This could be a problem only then if the total output of the university over various types is summed up. Quality control is organized at central levels within the universities using METIS (either by staff members of academic affairs departments or university libraries). Over the last few years, commercial parties such as Thomson Reuters and Elsevier have entered the market of CRIS systems, with respectively CONVERIS (originally developed by Avedas, a German company located in Karlsruhe, see http://thomsonreuters.com/converis/), and PURE, developed by the Danish company Atira, located in Copenhagen, and nowadays owned by Elsevier Science (http://www.elsevier.com/online-tools/research-intelligence/products-and-services/pure). CONVERIS, owned by Thomson Reuters, is a research information management system that allows the management of the full research life cycle, from the moment research grants are written and filed, to the end results stemming from the research grants, and the way these results are received by the community. Part of the functionality of CONVERIS consists of the registration facilities for research outputs, which is supported by access to both PubMed and Web of Science database. This is particularly helpful for those scientific disciplines where journal publishing is the standard.

In METIS, one can include various types of scientific output, which follows prescribed ways to register publications. In most bibliometric analyses, the focus is mainly on journal literature due to the focus on application in fields in which bibliometric techniques have a certain degree of validity (such as the natural, life and medical sciences), and bibliometrics is more accepted as a tool to assess the scientific performance and impact of domains under study (as described above). However, METIS contains a richness of information when one wants to analyze the output of domains outside the natural, life, and medical sciences. The system offers the possibility to register peer reviewed as well as non-peer reviewed journal publications, books (either monographs or edited volumes), chapters in books, conference papers, but also reports, magazine contributions, theses, and case reports. We will talk about this in more detail in the next section. As the university under study has a strong focus on social sciences, humanities, and law, it is important that a wide variety of outputs is registered in METIS. A central role in the evaluation of social sciences, humanities, and law research is played by the concept of scholarly publishing. If taken form a very strict definition, scholarly publishing relates to those kind of outputs that are related to the more traditional forms of scientific communication, in which peer review plays an important role. Types of output one should think of in this respect are publishing in scientific journals, contributing to edited volumes, and monographs, all peer reviewed during production. All kinds of publication forms that are not subject of peer review are thus not classified under scholarly publishing. A less traditional approach to scholarly publishing is the broadening of the perspective, both on the knowledge production and communications dimension (“who produces what types of scientific communication forms, and under what conditions), and equally important, also on the receiving end of the knowledge production process (“for who are the scholarly activities and outputs intended?”). So important here is to make a distinction between the various parties playing a role in the communication process. In the latter approach, also the targeted audiences play a role in the communication process.

## Data and methods

### Data

The data in this study which forms the basis of this study, are extracted from the research information system METIS, that is applied in Dutch academic environments. It is important to stress that access to METIS and information derived directly from METIS was extremely important, as we did not want to work with an extract from that system, made for other purposes, but rather work with the output data registered on an annual basis over the period 2004–2009, as that indicates most clearly and directly what the domains under study conceive themselves as their complete and unfiltered output. Having said that, it remains true however that the way in which publications in METIS are attributed to a scientific category or faculty is by and large the choice of the organization itself.

In this study we wanted to measure the impact of the university under study. Therefore, a bibliometric study was conducted, for which the Web of Science, from here on WoS, was selected. The WoS is the internet version of the combined Citation Indexes, the Science Citation Index Expanded, the Social Sciences Citation Index, and the Arts and Humanities Citation Index. The WoS contains mainly journal literature, although expansions of the WoS are possible (with books, conference papers, etc.) within various forms of subscriptions. In our case, we worked with the journal based version. The WoS version used within CWTS covers the period 1981–2013 and the running year. The criteria for inclusion of journals are relatively well formulated: journals have to be internationally oriented, publish preferably in English, have to have a peer review system, and appear on a frequent basis. Furthermore, citation analysis within the own data systems of Thomson Reuters plays a role in deciding on inclusion or not. The version used in this study was still stored in SAS. It is a well-known fact that the coverage of the WoS database is best for the life, medical, and natural sciences, has a lesser coverage in the engineering sciences, and some of the social sciences (such as psychology, economics, and business), and a relatively poor coverage in some other social sciences (such as political science, public administration, and anthropology), and the arts and humanities domains (Moed 2004; van Leeuwen 2013).

The coupling of the publication output to the WoS database is based upon an algorithm that uses the key bibliographic information of the publications as registered in METIS (such as first author, initials, source title, publication year, volume, and page numbers). This is a procedure which in various iterations, with exclusion of parts of the bibliographic information available, tries to match as much as possible METIS publications to the WoS database. The results of this procedure, developed in hundreds of bibliometric analyses, are manually checked for accuracy.

In order to be able to indicate the adequacy of current data systems providing a basis for bibliometric studies (that is, involving citation impact analysis), we needed to create insight into the degree to which the output of the domains under study were processed within the WoS. This type of analysis is referred to as the external coverage, as it indicates the degree to which the output is published in journal literature, and in other sources, and as such indicates the relevance of a citation index for the assessment of the domains under study [similar analyses can be found in Ossenblok et al. (2014), Sivertsen and Larsen (2012)]. In a next step, building upon the previous, we focus on yet another element in this adequacy analysis, namely the internal coverage. This is determined by an analysis of the references given by the researchers in their WoS publications, in order to establish to what extent they refer themselves to WoS journal publications. A relative high degree of referring to WoS covered journal publications indicates a high relevance of journal publications for the communication process, and if observed, allows a strong(er) focus on journal publications in the assessment of the research conducted by the domains under study.

A next step will involve actual scientific impact analysis. In the first place we will analyze the publications processed through our standard procedure, but next to that we will conduct a so called non WoS analysis: all publications processed in METIS, and submitted to CWTS for analysis that are not processed for a journal included in the WoS will be analyzed by the level of impact it still can have, by analyzing whether these publications are cited by the journal literature processed for the WoS (Butler and Visser 2006).

### Methods

We calculate several indicators for the oeuvre of a research domain, as produced within the time-frame of the study (cf. Nederhof and Visser 2004). For a detailed description we refer to Moed et al. (1995), as well as Waltman et al. (2011a, b). The methodology of database construction and bibliometric analysis is partially based upon previous work by Garfield (1979), Martin and Irvine (1983), Narin and Withlow (1990) and Van Raan (1997).

A *first* indicator in the WoS based research performance analysis gives the total number of papers published by the research domain during the entire period (*P*). We considered only papers classified as *normal articles*, *letters* and *reviews*. Meeting abstracts, corrections, and editorials are *not* included. In a few cases, a paper is published in a journal for which no citation data are available, or that is not assigned to a WoS Journal Subject Category. These papers are not considered in the calculation of the indicators presented in the tables below.

The next indicator gives the total number of citations received, without (*C*) self-citations. A self-citation (sc) to a paper is a citation given in a publication of which at least one author (either first author or co-author) is also an author of the cited paper (either first author or co-author). As an indication of the self-citation rate we present the percentage of self-citations (*%Selfcits*), relative to the total number of citations received (sc/(C + sc)). The *fourth* indicator is the average number of citations per publication calculated while self-citations are not included (*MCS*).

A *fifth* indicator is the percentage of articles not cited during the time period considered (*%Pnc*), excluding self-citations.

Next, two international reference values are computed. A first value represents the expected citation rate of the subfields in which the research domain is active (*FCS*, the field citation score). Our definition of subfields is based on a classification of scientific journals into WoS Journal Subject Categories developed by Thomson Reuters. Although this classification is certainly not perfect, it was at present the only classification available in our WoS environment. The *FCS* takes into account both the type of paper (e.g., normal article, review, and so on), as well as the specific years in which the research domain’s papers were published. For example, the number of citations received during the period 2005–2010 by an *article* published by a research domain in 2005 in field X is compared to the average number of citations received during the same period (2005–2010) by all *articles* published in the same field (X) in the same year (2005). Self-citations are excluded from the computation of *FCS*. In most cases, a research domain is active in more than one subfield (i.e., journal category). In those cases, we apply various field impact scores, as related to the individual publications, the selection of the fields being determined by the journals the research domain has used to publish its’ research findings.

The second reference value presents the expected citation rate of the journals in which the research domain has published (*JCS*, the journal citation score). In calculating *JCS*, we used the same procedure as the one we applied in the calculation of *JCS*, with subfields replaced by journals.

When a journal is classified in multiple subfields, as happens frequently in the WoS, citation scores are computed as follows. Basically, a paper in a journal classified in N subfields is counted as 1/N paper in each subfield, and so are its *FCSm* scores, so this creates per individual publication an expected mean field citation score.

We then arrive at the most important indicators compare the number of citations per individual publication within the oeuvre of a research domain (*C*) to the two international reference values, namely the corresponding journal and field expected citation scores of individual publications (*JCS* and *FCSm*, respectively), by calculating the ratio for every single publication against both expected citation scores. Self-citations are excluded in the calculation of the ratios *C/FCSm* and *C/JCS*, to prevent that citation scores are affected by divergent self-citation behavior. Over all ratios of individual publications, we calculate a mean impact score, for both the fields as well as the journals in which the institute has published.

This overall field normalized impact indicator for the output is *MNCS*, the mean normalized citation score. As this indicator focuses on the broader environment of the group’s output, this indicator seems the most suitable indicator of the international position of a research domain. If the *MNCS* is above (below) 1.0, this means that the output of the research domain is cited more (less) frequently than an ‘average’ publication in the subfield(s) in which the research domain is active. The *FCSm* values of the individual publications constitute a *world subfield average* in a specific (combination of) subfield(s). In this way, one may obtain an indication of the international position of a research domain, in terms of its impact compared to a ‘world’ average. This ‘world’ average is calculated for the total population of articles published in WoS journals assigned to a particular subfield or journal category. As a rule, about 70–80 % of these papers are authored by scientists from the United States, Canada, Western Europe, Australia and Japan. Therefore, this ‘world’ average is dominated by the Western world.

A second important indicator, *MNJS*, is above 1.0 if the citation score of the journal set in which the research domain has published exceeds the citation score of all papers published in the subfield(s) to which the journals belong. In this case, one can conclude that the research domain publishes in journals with a relatively high impact.

The *MNCS/MNJS* indicator matches the impact of papers closely to the publication pattern of the journals selected for publication. If the ratio *MNCS/MNJS* is above 1.0, the impact of a research domain’s papers exceeds the impact of all articles published in the journals in which the particular research domain has published its papers (the research domain’s journal set). A limitation of this indicator is that low impact publications published in low impact journals may get a similar score as high impact publications published in high impact journals.

It should be noted that the *MNCS*, *MNJS* and the *MNCS/MNJS* indicators are not independent. The value of each one of these follows directly from the values of the other two indicators.

## Results

In this section we will present the results of our analyses. We will start with a description of the publication material CWTS analyzed from the university.

**Fig. 1:**
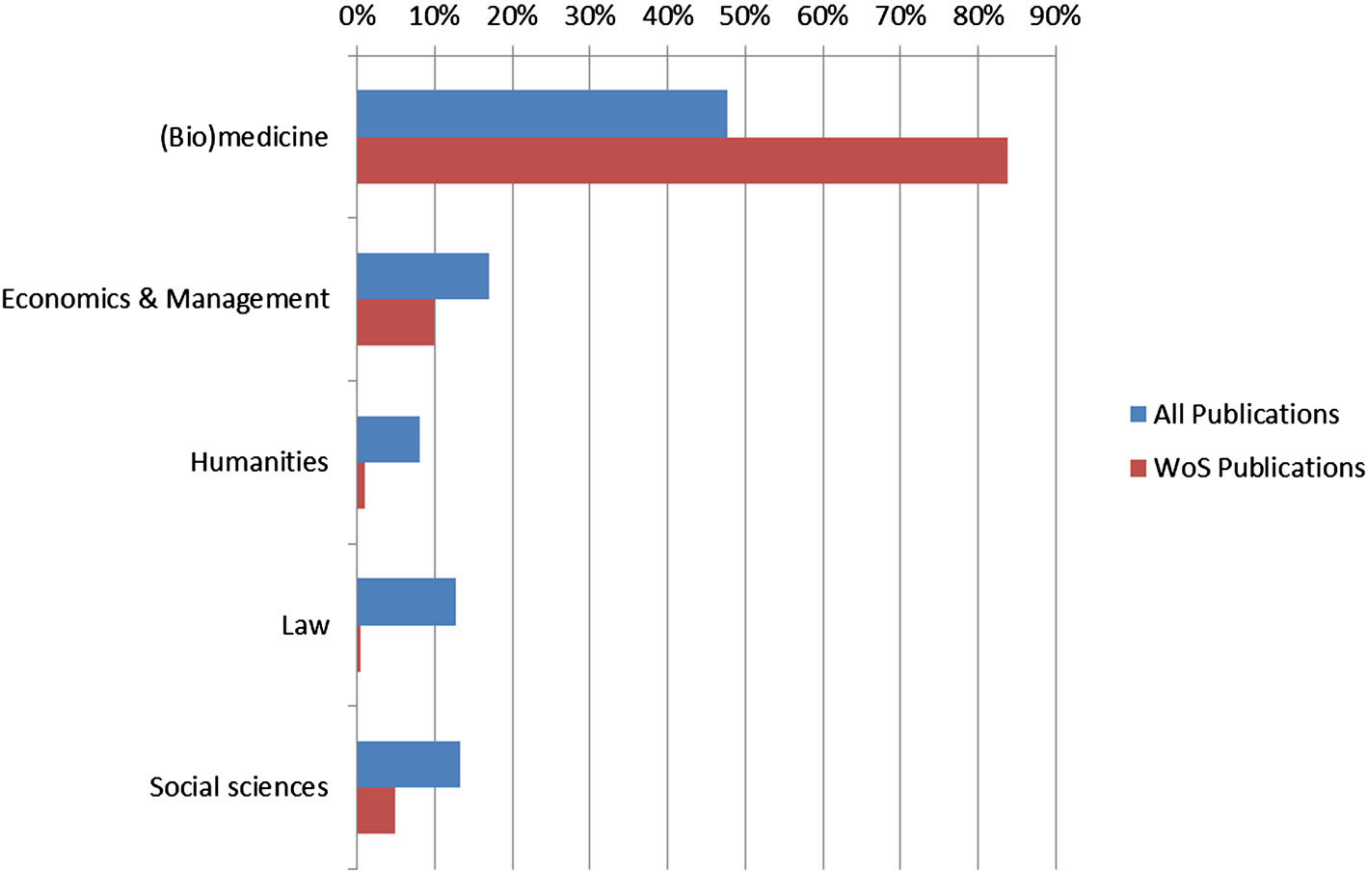
Comparing total output and WoS covered output for domains, 2004–2009

In Table 1, the results are shown for the input in the study. The first column, *Inputcount* indicates the total number of publications submitted in METIS and as such input to the study. In total 48 % of all submitted publications are from the (Bio)medicine domain, while Economics and management cover the second largest output registered in METIS with 18 % of the output, followed by Law and Social sciences which each cover 13 %. The Humanities cover 8 % of the total registered output. After matching with the WoS (resulting in the variable *Matchcount*), we notice that the dominant position of (Bio)medicine domain has increased strongly, as the total number of publications from that domain has risen to 84 % of all publications. This is graphically underlined in Fig. 1, illustrating the dominant situation of (Bio)medicine when it comes to WoS covered journal publications.

**Table 1:** Overview of input and output of CWTS matching procedures for output data in METIS, 2004–2009

	Input count	%	Match count	%
(Bio)medicine	18,807	48	12,950	84
Economics and management	6902	18	1485	10
Humanities	3128	8	164	1
Law	4995	13	65	0
Social sciences	5238	13	750	5
	35,296		14,093

Table 2 presents the composition of the university’s output over various types of publication types as used in METIS. We find books and book chapters (*Book* and *Chap*), cases (or case studies, which has a different connotation in either the (Bio)medicine and Law domain, indicated as *Case*), Conference papers (*Conf*), Journal publications (*Jour*), Magazine contributions (*Mgzn*), Patents (*Pat*), reports (*Rprt*) and such like. The only category somewhat unclear is the category functioning as a container (*Gen*). It is important to unravel this category as it contains a substantial number of publications, and thus an important share of the output for some domains (Social sciences and the Humanities).

**Table 2:** Composition of domains over various types of publication, 2004–2009

	(Bio)medicine	Economics and management	Humanities	Law	Social sciences
BOOK	1.1	3.9	7.6	6.0	5.5
CASE	0.0	0.5	0.0	15.3	0.0
CHAP	8.1	13.6	22.7	24.0	17.1
CONF	0.0	9.0	0.3	0.9	2.1
GEN	0.3	7.6	31.1	9.2	23.7
JOUR	86.8	43.3	30.5	40.9	36.8
MGZN	0.1	2.9	6.9	0.9	5.9
PAT	0.3	0.0	0.0	0.0	0.0
RPRT	0.9	16.4	0.5	1.3	8.2
THES	2.2	2.8	0.4	1.5	0.7

**Fig. 2:**
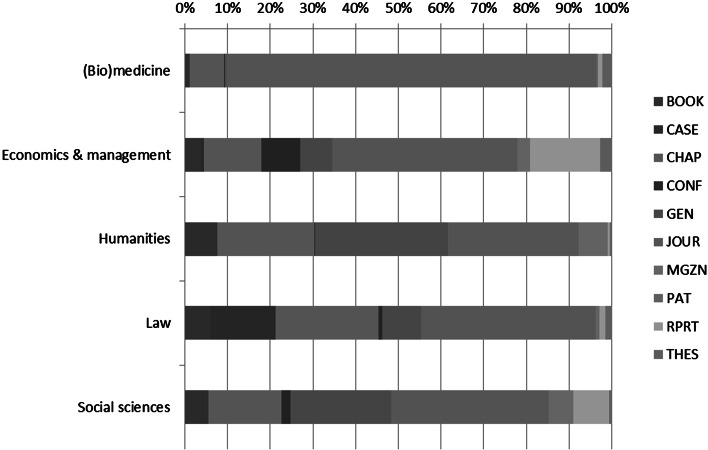
Composition of the output for domains, 2004–2009

Figure 2 graphically displays Table 2, and it becomes immediately clear that journal publications are most important for (Bio)medicine, although not unimportant for the other domains in the university. It becomes clear that the other domains do publish in journals, the most remarkable fact here is that they do not publish in WoS covered journals, as can be concluded from analyzing Figs. 1 and 2. Books and book chapters are very important for particularly the Social sciences and the Humanities, and to a lesser extent for Economics and management. Cases are important for Law, this category contains annotations to current law practice, while Reports are important for Social sciences, and Economics and management. Finally, both (Bio)medicine and the Economics and management have over 2 % of their output in Theses.

Next, comparing the outcomes of Fig. 1, where the visibility within the WoS is indicated, in combination with Fig. 2, in which the variety in scientific outputs is displayed, we come to the conclusion that other types of scientific communication and publishing (such as professional journal publications, magazines, newspaper contributions, etc.), which are often considered as non-scholarly output, are of more importance for most domains in the university, whereas WoS covered literature is most important for the (Bio)medicine domain (the traditional scholarly outputs).

In Table 3, we present the results of the internal coverage analysis, that is, an analysis of the references given by the researchers writing the publications covered by WoS. This analysis starts from the hypothesis that referring to WoS publications indicates some sort of relevance for your community, so whether or not WoS journals do play a role in communicating findings, and as such can be interpreted as an assessment of the adequacy of the relevance of WoS in evaluating research performance of domains or fields. Table 3 clearly indicates the high degree of relevance of (WoS) journal literature for (Bio)medicine (as the *%refs CI* is nearly 90 %), although the average length of reference lists (*Avg Total Nr Refs*) of researchers of other domains is equally long or even longer. So the WoS journal papers of the other domains contain many references, but not only to WoS publications themselves. Somewhat higher levels of coverage are observed for the Social sciences, and Economics and management, while the lower levels of coverage of WoS journal literature are found for the Humanities and Law).

**Table 3:** Internal coverage of domains through WoS publications, 2004–2009

	P 04-09	Avg total Nr refs	%Refs < 1980	Nr refs > 1979	%Refs CI
(Bio)medicine	12,045	36.44	3	425,435	89
Economics and management	1381	43.76282	8	55,375	62
Humanities	105	41.39667	19	3517	30
Law	47	39.23	10	1654	46
Social sciences	671	43.3	7	27,015	61

**Fig. 3:**
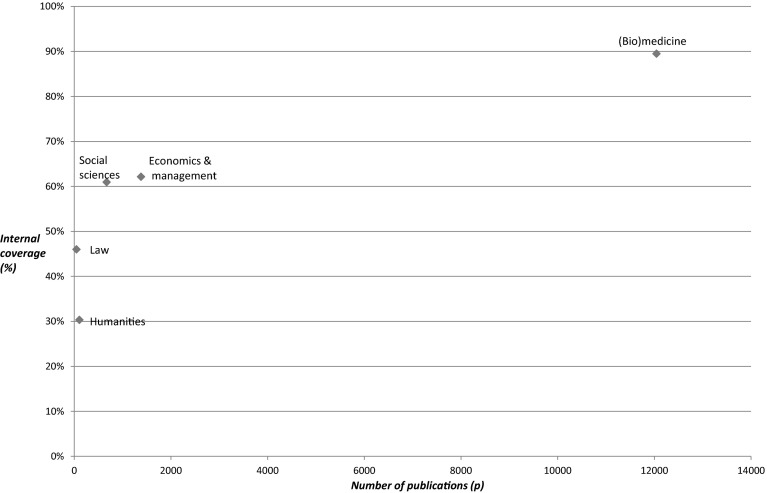
Output for domains compared to coverage assessment in WoS, 2004–2009

In Fig. 3, the (sharp) differences in both output numbers as well as in the degree of relevance of WoS (as expressed through reference analysis) are illustrated. WoS is most relevant for (Bio)medicine, while the Social sciences, and Economics and management come next with percentages around 60 %, followed by the other two domains where WoS publications are clearly of lesser importance in the scholarly communication process, given the shares of references given by their scientists towards other WoS covered literature.

Next, In Table 4 we present the results of the impact analysis in WoS publications for the domains of the university. As stated before, most publications in WoS are observed for (Bio)medicine, and the related field normalized impact is high as found in previous analyses for this domain. More interestingly in this study is the focus on the international visibility and impact of the other domains. The social sciences related domains (Social Sciences, and Economics and management) with nearly 700 and 1400 publications receptively in WoS journals, display impact levels that are well above worldwide average impact level. The average impact per publication is lower, a reflection of the differences in citation practices between these two domains and (Bio)medicine. Most domains display a high percentage of publications not cited within the time frame of the period 2004–2010 (as the citation window was stretched to contain 2010), which is also a reflection of the publication and citation practices in the fields in which the other domains are active. Remarkably enough, a high impact is found for the Humanities. The 113 publications get cited on average three times, but compared to the field that stands out as MNCS (the field normalized impact indicator) indicates an impact level of 1.85, while the publications appeared in top journals in the field to which these journals belong (as indicated by the MNJS value of 1.73). Furthermore, the visibility among the top 10 % most highly cited publications also indicates that this is not only based on only one or a small number of publications.

**Table 4:** Bibliometric statistics of domains for WoS publications, 2004–2009/2010

	*P*	C (excl sc)	mcs	mncs	mnjs	mncs/mnjs	Pnc (%)	%Self citations	Ptop10 %
(Bio)medicine	11,742.3	263,984.0	22.48	1.61	1.38	1.17	4	18	18
Economics and management	1401.8	14,187.3	10.12	1.41	1.30	1.08	10	17	18
Humanities	113.3	344.8	3.04	1.85	1.73	1.07	24	25	17
Law	50.0	262.0	5.24	0.80	1.19	0.67	18	17	8
Social sciences	688.0	7646.0	11.11	1.44	1.15	1.25	7	21	17

**Fig. 4:**
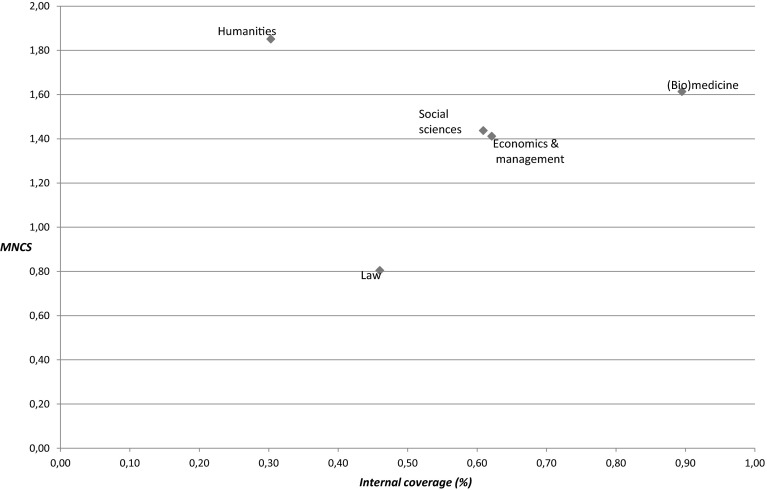
Impact compared to percentage coverage for domains, 2004–2009

Figure 4 further underlines that low (internal) coverage (that is, a low focus on WoS journals) not automatically means a low impact whenever these journals are selected as communication medium, as the Humanities do have a very high impact in comparison with the relevance of WoS journals for this domain.

## Non WoS citation analysis of domains in the university

In this section, the results of the non WoS citation analysis are presented. This citation analysis is conducted in the realm of the WoS itself, so the citations given in WoS journals towards sources not processed for the WoS themselves. This means that the results probably present the tip of the iceberg, as in sources of the same kind more citations could be found, unfortunately we do not have these available for citation analysis.

Table 5 contains the publication data that are not processed for the WoS. So the second column presents all publications not found to be WoS covered papers. The third column contains publications that are not cited within the realm of the WoS, while the fourth column contains the publications cited within the journal literature processed for the WoS in the period 2004–2010. The fifth column contains all citations received by non WoS covered publications, while the final two columns present the average impact scores for these types of publications, the sixth column presents the overall average, while the seventh column shows the mean impact for the cited publications only. As we have calculated a full average for the WoS part as well, the sixth column contains the actual CPP value as we also calculated for the WoS covered output of university domains.

**Table 5:** Results of the non WoS citation analysis for domains, 2004–2009

	*P* non WoS	%*P* non WoS	*P* non cited non WoS	*P* cited non WoS	%*P* cited non WoS	Cits	CPP + sc all	CPP + sc cited only
Hospital	5857	31	4989	868	15	4514	0.77	5.20
Economics and management	5417	78	5088	329	6	2197	0.41	6.68
Humanities	2964	95	2901	63	2	104	0.04	1.65
Law	4930	99	4871	59	1	97	0.02	1.64
Social sciences	4488	86	4277	211	5	961	0.21	4.55

As we clearly observe in the non WoS citation analysis, the mean impact is very low for all the domains. This is further underlined by making the comparison with the WoS covered output of the university, which is done in Table 6. Here it becomes immediately clear that the additional non WoS citation analysis for all domains is hardly contributing to an improved international visibility of the various parts of the university. However, focusing on the citations received by the cited publications only clearly show that some domains do get cited in the international serial literature as covered by WoS, as the Humanities do get cited nearly as often on this particular part of their research output, as compared to the WoS covered output parts (see also Nederhof et al. 2010). A next conclusion could be that those domains that have already a high visibility in the WoS covered journal literature have a relative advantage, as their journal publications are already known in this realm, and so will their non WoS covered sources be probably better known among the scholars publishing in the WoS covered journal literature.

**Table 6:** Comparing mean citation impact scores for WoS and non WoS publications of domains, 2004–2009

	CPP WoS papers	CPP non WoS papers	CPP cited non WoS papers
(Bio)medicine	22.48	0.77	5.20
Economics and management	10.12	0.16	3.98
Humanities	3.04	0.04	2.47
Law	5.24	0.02	1.64
Social sciences	11.11	0.41	6.68

## Conclusions

This study, which starts from the perspective of the output of a Dutch university as a whole and the constituting domains (five in total), clearly indicates the possibilities and limitations of the bibliometric methodology as it is right now. The study leads to a number of conclusions on various topics dealt with in the study:

- The (low degree of) validity of bibliometric techniques in domains of the social sciences, humanities and law;
- The importance of *External/Internal coverage analysis*, particularly in the social sciences, humanities and law;
- The importance of covering a wide variety of different types of scientific communication;
- The dependence on the quality of the input into a Research Information System such as METIS;
- The way the various categories in METIS are defined and/or filled;

The study has made very clear that a sole dependence on bibliometric techniques as can be applied with ease in the medical, life and natural sciences, and with some effort in the engineering sciences, mathematics, statistics and some social sciences (economics, psychology, management science), do not work properly in the other social sciences, the humanities and law research. The publication culture is clearly focused on different sources of scientific communication, sources that are not processed for the Web of Science (or Scopus, for that matter), such as books, book chapters, cases, and still, to a large extent, journals that are not processed for the WoS or Scopus. Many of these journals not processed for the WoS do appear in other languages than English, many of them in the own Dutch language. A typical example of the different scientific tradition or publication culture in the social sciences, humanities and law is the fact that the category *General* in METIS is filled by researchers in these disciplines by activities (coordinator, committee member, membership of editorial board), next to the more classical registration of scientific activities and codification of knowledge that takes place in the natural, life and medical sciences, namely through journal publications.

The study is a clear example of the comparison of the validity of the usage of WoS based bibliometric techniques, as it explores to the fullest extent the way the output of the various parts of the university is covered by WoS. By both an External coverage analysis (how is the output composed, what part of the output is WoS covered), and an Internal coverage analysis (to what extent do scholars in the various parts of the university refer to scientific sources, and to what extent do WoS covered journal play a dominant role in that referencing behavior, indicating the relevance of WoS based analyses for these domains under study), the study supplies the reader with a clear insight into the relevance of these WoS based bibliometric techniques for the various parts of the universities under study. As we expected, the WoS based methodology works very good for the medical domain, works relatively well for the social sciences, management and economics based part of the university we studies, but is weak when it comes to the humanities and law parts of the university in this study.

The study provides a clear insight into the various types of scientific communication used by the different parts of the university studied. We clearly notice the relevance of books and book chapters for the humanities and law domains, as well as a strong focus on non WoS covered journal literature, often locally oriented and not in English.

An important topic in this discussion on the scientific impact and relevance of the scientific activities of researchers in these fields relates to the topic of social or societal impact or relevance. It seems that whenever the scientific impact cannot be measured directly or is somewhat problematic, societal impact becomes the replacing magic trick in order to cover the activities of researchers in these fields. This might be a conclusion drawn to fast. It should be possible to create a common ground between researchers in a field on how to measure the scientific impact or relevance of work done by academics, while the discussion on societal impact is more far-reaching, as scientific impact can be seen to have societal relevance as well. This will not be solved in this study; this only describes the landscape in which this development is placed.

The study has shown the dependence on the input in a Current Research Information System such as METIS. Although relatively standardized by design, the weak part here is the dependence on the way the data are entered into the system. Although a general problem (this is namely also the case for the WoS), this is more problematic for METIS, as local guidelines might lead to quite some variation in the way publications are processed for registration in METIS. Some frequent occurring problems are the possibilities of double counts (publications entered into METIS in one university in two different ways, or in case of scientific cooperation, in two different ways at two universities), the way a category such as *General* becomes a container of various types of scientific activity, thereby diluting the way some categories are filled (e.g., the inclusion of local journal publications or magazine like publications in *General*, while for both types a specific category exists). Here the disciplines under study could improve their visibility, by having better protocols for entering publication data into METIS (or METIS like systems).
